# Efficacy of Acetazolamide for Treatment of Iatrogenic, Traumatic, and Spontaneous Cerebrospinal Fluid Leaks of the Anterior Skull Base: A Systematic Review

**DOI:** 10.7759/cureus.75214

**Published:** 2024-12-06

**Authors:** Spencer L Raub, Zachary A Abecassis, Thomas A Hanks, Kyly Hiatt, Aria Jamshidi, Emma Celano, Manny Ferreira, Sam Emerson, Jacob Ruzevick

**Affiliations:** 1 Neurological Surgery, University of Washington Medical Center, Seattle, USA; 2 Neurological Surgery, University of Washington Medical Center, Seatle, USA

**Keywords:** acetazolamide, diamox, endoscopic csf leak, spontaneous csf leak, traumatic csf leak

## Abstract

A cerebral spinal fluid (CSF) leak from the anterior skull base is a challenging neurosurgical issue that requires prompt recognition and treatment. Options for treatment include medical and surgical repair. A systematic review was performed screening for both retrospective and prospective clinical studies evaluating the efficacy of acetazolamide in the event of CSF leaks of the anterior skull base.  We initially screened a total of 149 studies for inclusion, and 25 of them met the inclusion criteria. We included four studies on iatrogenic CSF leaks caused by surgery of the anterior cranial fossa, three studies on traumatic CSF leaks, and 18 studies on spontaneous CSF leaks caused by idiopathic intracranial hypertension (IIH). In the event of an iatrogenic CSF leak, 68 patients had undergone an endoscopic endonasal approach. CSF diversion and high-volume lumbar puncture were used frequently with adjunct acetazolamide administration. In the event of trauma, 187 patients were evaluated across the three included studies. Acetazolamide treatment was used as a single approach and effectively controlled CSF leaks. For spontaneous CSF leaks, acetazolamide is frequently used to increase the efficacy of surgical repair. Of the 431 patients, 327 received temporary CSF diversion in addition to acetazolamide. Surgical repair was the primary treatment in 277 patients. Acetazolamide should be considered as a treatment option in patients with CSF leak secondary to surgery of the anterior cranial fossa, trauma, and idiopathic causes.

## Introduction and background

Cerebrospinal fluid (CSF) leakage from the anterior skull base is a complex issue that requires prompt intervention. This can be a result of trauma (typically in the event of a complex facial or sinus fracture), iatrogenic (from a prior craniotomy or transsphenoidal operation), or chronic skull erosion from intracranial hypertension [1]. A broad array of options within a neurosurgeon armamentarium include surgical repair via a transcranial or endonasal approach, medical treatment of elevated intracranial pressure, and/or permanent CSF diversion [2]. Unfortunately, CSF leaks can recur in the event of a suboptimal reconstruction, necrosis of a pedicled nasoseptal or pericranial flap, or if underlying risk factors, such as intracranial hypertension, are not addressed [3]. This can lead to persistent morbidity, including headaches and, in severe cases, meningitis. Surgical repair is often necessary in this setting to correct the underlying defect [4,5]. Medical therapies have been suggested as both a primary and a supplementary treatment for CSF leak, as they may improve surgical efficacy, reduce recurrence, or, in rare cases, circumvent the need for surgery altogether [6-8].

Acetazolamide is a carbonic anhydrase inhibitor that is predominantly prescribed within the neurosurgical and skull base communities for its use in lowering intracranial pressure in patients with idiopathic intracranial hypertension (IIH) [9]. Its use for the treatment of CSF leaks has been well described, but its exact indication and efficacy are variable [10,11]. To date, there is little consensus on the use of acetazolamide for the treatment of CSF leaks beyond its use in IIH. Therefore, our objective with this systematic review is to provide a comprehensive assessment of the efficacy of acetazolamide in the treatment of iatrogenic, traumatic, and high-pressure CSF leaks of the anterior skull base.

## Review

Protocol and eligibility criteria 

This systematic review was conducted following the PRISMA guidelines. Studies were included if they met the following criteria: (i) peer-reviewed articles, (ii) published in English, (iii) involved human participants, (iv) investigated anterior skull base CSF leaks and use of acetazolamide, and (v) utilized prospective/retrospective studies, cohort studies, case studies, or RCTs. Exclusion criteria were studies that did not discuss anterior skull base CSF leak and acetazolamide use together, non-original research such as editorials, reviews, or studies lacking sufficient data or where the full text was inaccessible.

Information sources 

A comprehensive search was performed across PubMed and Embase. The search was conducted without time or geographical restrictions. Reference lists of included studies and relevant systematic reviews were screened to identify additional eligible studies.

Search strategy 

The search strategy was developed using a combination of medical subject headings (MeSH) and free-text terms related to anterior skull base CSF leaks and acetazolamide use. The search terms were tailored for each database. For example, the PubMed search included terms such as: (‘acetazolamide,’ OR ‘Diamox,’) AND (‘cerebrospinal fluid leak,’ OR ‘CSF leak,’ OR ‘post-operative CSF leak,’ OR ‘endoscopic transsphenoidal surgery CSF leak,’ OR ‘anterior skull base CSF leak,’ OR ‘traumatic CSF leak.’).

Study selection 

**Figure 1:**
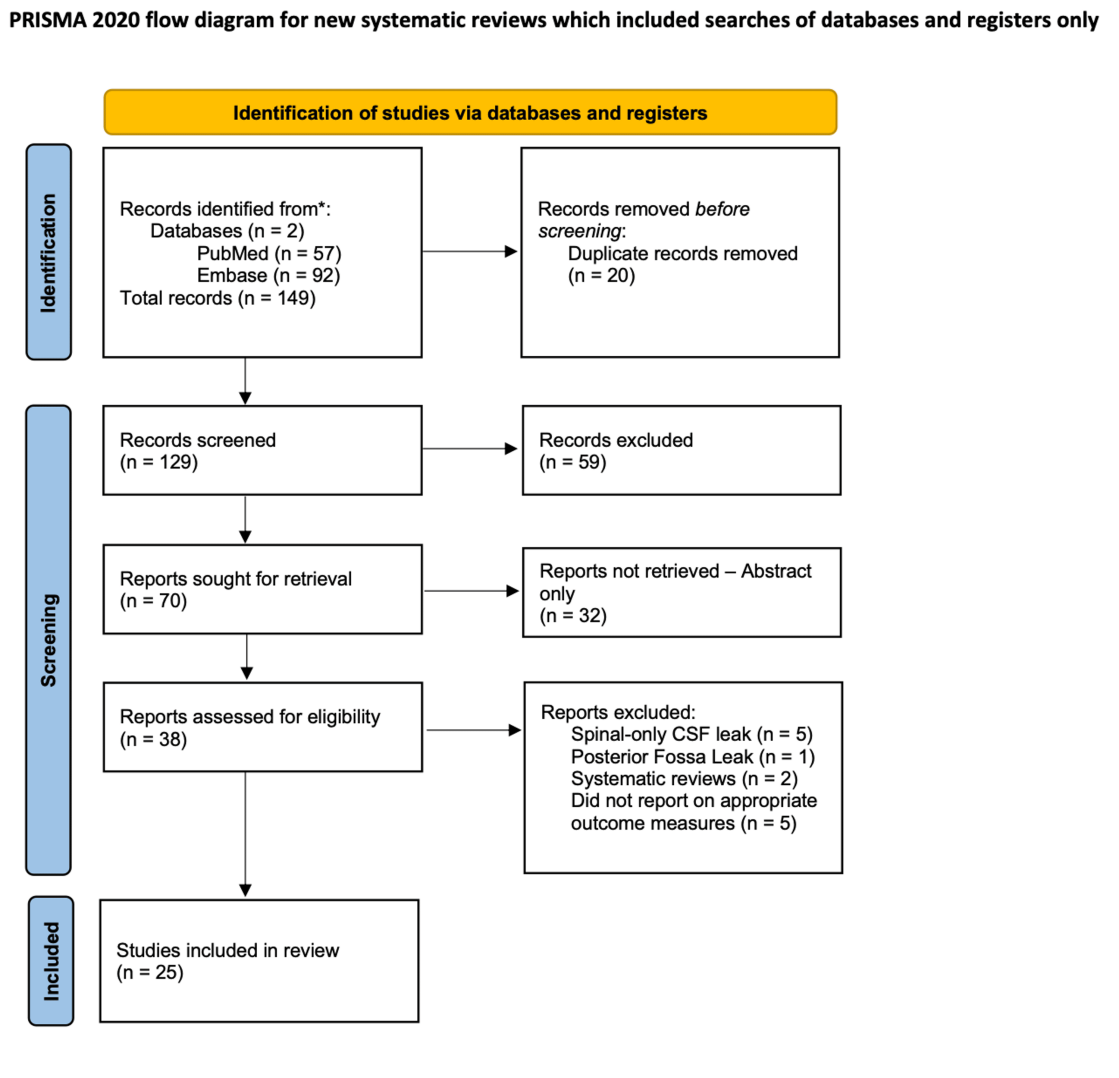
PRISMA flow diagram indicating inclusion and exclusion criteria for selected studies Source [12]

Two independent reviewers screened titles and abstracts based on the eligibility criteria. Full-text articles of potentially relevant studies were retrieved and assessed for inclusion. Disagreements between reviewers were resolved through discussion or by consulting a third reviewer. The study selection process was documented using a PRISMA flow diagram (Figure 1) [12].

Data extraction 

Data were extracted independently by two reviewers using a standardized extraction form. The extracted data included study characteristics (author, year, study type), demographics, interventions, comparators, and outcomes. Discrepancies in data extraction were resolved through discussion or consultation with a third reviewer. If necessary, authors were contacted for missing or unclear data.

Risk of bias 

Two reviewers independently assessed the risk of bias for included studies using the Cochrane Risk of Bias tool for randomized trials and the Newcastle-Ottawa Scale for cohort studies (Tables 1-2). Discrepancies in risk of bias assessments were resolved through discussion or by involving a third reviewer [13-31].

**Table 1:** Risk of bias assessment for two randomized controlled trials evaluating acetazolamide in CSF leakage management This table assesses the risk of bias in two randomized controlled trials (RCTs) using the Cochrane risk of Bias (RoB 2) tool. It evaluates five key domains: the randomization process, deviations from intended interventions, missing outcome data, measurement of the outcome, and the selection of the reported results. "High risk" indicates a significant concern for bias, "Low risk" suggests minimal bias, and "Unclear risk" denotes insufficient details to assess the bias.

Domain	Abrishamkar et al. [18]	Madeshiya et al. [10]
Randomization process	High risk: the study lacks details on how randomization was performed, and allocation concealment was not mentioned, raising concerns about potential selection bias.	Low risk: described as a randomized controlled trial with an appropriate randomization process. Allocation concealment is well-documented.
Deviations from intended interventions	Unclear risk: the study does not provide clear details on the blinding of participants and personnel, making it uncertain whether deviations occurred.	Low risk: proper blinding of participants and researchers was implemented, minimizing deviations from the intended interventions.
Missing outcome data	Low risk: no significant missing outcome data was reported, and reasons for participant exclusions were clearly explained.	Low risk: all participants accounted for with complete follow-up data, with reasons for any exclusions transparently reported.
Measurement of the outcome	Unclear risk: no mention of whether the outcome assessors were blinded, introducing a potential risk of bias in outcome measurement.	Low Risk: outcome assessors were blinded, reducing the potential for bias in the measurement of outcomes.
Selection of the reported result	High risk: the study appears to have selectively reported outcomes, with no reference to a pre-published protocol or registration of the trial.	Low risk: outcomes were reported as pre-specified in the study protocol, and the trial was registered, minimizing selective reporting bias.

**Table 2:** Newcastle-Ottawa Scale bias analysis for studies evaluating acetazolamide on CSF leak management This table provides a bias assessment for studies on cerebrospinal fluid (CSF) rhinorrhea and related management strategies using the Newcastle-Ottawa Scale (NOS). The NOS evaluates studies across three domains. Selection (0-4 stars): Measures the representativeness of the study groups and the selection process. Comparability (0-2 stars): This rating assesses the comparability of study groups, particularly the control of confounding variables. Outcome (0-3 stars): Focuses on the quality of outcome assessment and the adequacy of follow-up. A total score out of 9 is given, with higher scores indicating a lower risk of bias. Studies with scores of 8 or 9 are considered to have a low risk of bias, while those with scores of 6 or 7 are considered to have a moderate risk of bias. This scale helps determine each study's overall quality and reliability.

Study	Selection (0-4)	Comparability (0-2)	Outcome (0-3)	Total (0-9)	Bias assessment
Kreatsoulas et al. [1]	★★★	★★	★★★	8/9	Low risk of bias; strong methodology.
Jiang et al. [2]	★★★★	★★	★★★	9/9	Low risk of bias; excellent across domains.
Chaaban et al. [5]	★★★	★★	★★★	8/9	Low risk of bias; well-designed study.
Tilak et al. [8]	★★★	★★	★★	7/9	Low risk of bias; robust but lacks some outcome detail.
Woodworth et al. [11]	★★★	★★	★★	7/9	Low risk of bias; lacks some comparability.
Jamshidi et al. [13]	★★★	★★	★★★	8/9	Low risk of bias; solid methodology.
Phogat et al. [14]	★★★★	★★	★★	8/9	Low risk of bias; well-designed.
Jaman et al. [16]	★★★	★★	★★	7/9	Moderate risk of bias; lacks robust outcome data.
Leibu et al. [17]	★★★	★★	★★★	8/9	Low risk of bias; strong outcome assessment.
Chaaban et al. [19]	★★★	★★	★★	7/9	Moderate risk of bias; lacks some outcome detail.
Jiam et al. [15]	★★★★	★★	★★	8/9	Low risk of bias; strong selection process.
Brainard et al. [20]	★★★	★★	★★	7/9	Moderate risk of bias; lacks detailed outcome reporting.
Martínez-Capoccioni et al. [21]	★★★	★★	★★	7/9	Moderate risk of bias; outcome assessment unclear.
McCormick et al. [23]	★★★★	★★	★★	8/9	Low risk of bias; strong methodology.
Martínez-Capoccioni et al. [24]	★★★	★★	★★	7/9	Moderate risk of bias; lacks some outcome assessment clarity.
Aaron et al. [25]	★★★	★★	★★	7/9	Moderate risk of bias; lacks some outcome data.
Hong et al. [26]	★★★★	★★	★★★	9/9	Low risk of bias; excellent across all domains.
Alkhotani et al. [27]	★★★	★★	★★	7/9	Moderate risk of bias; lacks detailed follow-up information.
Matsubara et al. [28]	★★★	★	★★	6/9	Moderate risk of bias; less robust comparability and outcome data.
Al-Balushi et al. [30]	★★★	★★	★★	7/9	Moderate risk of bias; some issues with outcome follow-up.
Lin et al. [31]	★★★	★★	★★	7/9	Moderate risk of bias; lacks robust comparability of groups.

Outcomes and synthesis of results 

The primary outcome of interest was CSF leak recurrence. Secondary outcomes included the rate of permanent CSF diversion and the need for surgical intervention. A narrative synthesis was performed for studies deemed too heterogeneous for meta-analysis. Subgroup analyses were conducted to explore potential sources of heterogeneity. Subgroup analyses were performed based on population characteristics (iatrogenic, traumatic, or elevated ICP-related skull base CSF leaks), and sensitivity analyses were carried out to assess the robustness of the results by excluding studies at high risk of bias.

Results 

In total, 149 studies resulted when the Boolean operators were applied with the MeSH query in PubMed and Embase. After the initial review, the exclusion criteria led to the exclusion of 124 studies from further review. Thirty-two articles were available in an abstract-only format and were not reviewed further. Twenty studies were duplicate articles and excluded from further review. Further review revealed that 64 articles did not meet the inclusion criteria, leading to their exclusion from the analysis. Five articles only analyzed spinal CSF leaks and one article only analyzed posterior fossa CSF leaks and were subsequently excluded, as well as two articles that were systematic reviews of CSF leaks and IIH. The remaining 25 studies met the inclusion criteria following review and were included for further analysis. 

In the 25 articles included in our review, acetazolamide was used in the treatment of CSF leaks resulting from surgery, trauma, or high-pressure CSF leaks secondary to IIH. Four articles covered its use as a primary or adjunctive treatment for CSF leak from a surgical procedure. Three articles described the role of acetazolamide as a primary treatment in managing CSF leaks secondary to traumatic injury, while the remaining eighteen articles discussed the use of acetazolamide as a primary or adjunctive treatment for spontaneous CSF leaks in the setting of IIH.

Acetazolamide for Treatment of Iatrogenic CSF Leak

There were four studies (one prospective, two retrospective, and one case report) that evaluated the use of acetazolamide for a CSF leak following surgery [13-16]. All of these studies were post-endoscopic endonasal procedures where the patients subsequently presented with CSF rhinorrhea [13-16]. The follow-up periods varied from six months to 3.9 years. Most of these patients underwent temporary lumbar drain placement during their admission (49/68 patients across all four included studies). One center performed a high-volume lumbar puncture of 30-40 ccs for the four patients that had CSF leaks. In one study, 12/53 patients had their CSF leak resolved with acetazolamide and head-of-bed restrictions alone. The remainder (41/54) eventually required lumbar drain placement [14]. The dose of acetazolamide was not reported in three of the four cases. One study reported a dose of 250 mg three times daily [13]. The range for eventually needing re-operation for persistent CSF leaks was 0% to 90%. The need for additional interventions was not consistently documented, but one study documented a 30% recurrence rate [15]. Only one of the 68 patients in these studies required permanent CSF diversion [15].

Acetazolamide for Treatment of Traumatic CSF Leak

There were three studies (two prospective and one retrospective) with a total of 187 patients evaluating the efficacy of acetazolamide in the event of traumatic CSF leaks [10,17,18]. Two of the three studies included follow-up, which ranged from 4 to 18 months. Leibu et al. evaluated 54 patients with CSF leaks after trauma within the pediatric population and found 47 out of 54 patients had their CSF leak resolved with acetazolamide treatment without surgical intervention. The study did not report on the dosing of acetazolamide [17]. Of the seven that failed initial conservative management with acetazolamide, three improved with additional lumbar drainage alone, and four eventually required surgical intervention. Abrishkama et al. evaluated the timing for acetazolamide administration by randomizing two cohorts of patients based on timing (pre- and post-48-hour mark from CSF leak) [18]. In the cohort started on acetazolamide within the first 48 hours (28/57), 26 patients had their leak resolve with standalone medication, whereas two eventually required lumbar drainage and one required surgical intervention. Of the cohort with delayed administration, nine of the 29 patients required lumbar drainage, and three eventually required surgical intervention [18]. In a second prospective study of 76 patients by Madeshiya et al., two cohorts of patients were evaluated, with one cohort comprised of 44 patients receiving a daily dose of 15 mg/kg of acetazolamide and another cohort that was observed without intervention [10]. In this study, patients who underwent temporary CSF diversion or required upfront repair were excluded. Notably, this study found a non-significant difference in the duration of CSF leak between those who did and did not receive acetazolamide. The study's exclusion criteria did not document any outcomes regarding the need for surgical intervention or CSF diversion.

Acetazolamide for Treatment of Spontaneous CSF Leak

There were 18 studies included for further review that highlighted the treatment of spontaneous CSF leaks with acetazolamide in the setting of IIH [1,2,5,8,11,19-31]. Tilak et al. analyzed the use of acetazolamide as a primary treatment in 16 patients with an average follow-up of 470 days. Of these 16 patients, 11 (68.6%) did not have resolution of the CSF leak and required subsequent surgical repair. Post-operative recurrence of the CSF leak was not reported [8]. The other 17 studies, which included 431 patients, analyzed the use of acetazolamide as an adjunct to surgical repair of the leak with a range of follow-up time from 5.4 months to six years (Table 3). Nine of these studies were retrospective, three were prospective, and five were case series/reports. In these studies, acetazolamide was utilized as an adjunct to repair in 277 of 431 patients, and on average most patients (75.8%) also underwent temporary CSF diversion with either a lumbar drain or an external ventricular drain regardless of acetazolamide use. Notably, two case studies reported that when temporary CSF diversion was used in conjunction with acetazolamide, permanent CSF diversion was not necessary [28,31]. Additionally, Alkhotani reported that their patient required permanent CSF diversion when they used acetazolamide without temporary CSF diversion [27].

**Table 3:** Demographics and outcomes for articles included for systematic literature review of acetazolamide in the treatment of iatrogenic, traumatic, and spontaneous CSF leaks AZM: acetazolamide; CSF: cerebrospinal fluid; ETSS: endoscopic transsphenoidal surgery.

Author	Year	Study type	CSF leak type	# of patients	Average follow-up time	Rate of management with AZM	AZM as primary or adjunct therapy	Rate of temporary CSF diversion and type	Rate of permanent CSF diversion	Need for surgical intervention	AZM dosing	CSF leak recurrence rate
Jamshidi et al. [13]	2022	Prospective	Iatrogenic ETSS	4	154.75 days	4/4 (100%)	Primary	100%	0%	0%	250mg TID x 10 days	0%
Phogat et al. [14]	2021	Retrospective	Iatrogenic ETSS	53	Not Reported	53/53 (100%)	Primary	77%	0%	6/53 (11.3%)	Not Reported	Not Reported
Jiam et al. [15]	2021	Retrospective	Iatrogenic ETSS	10	3.9 years	1/10 (10%)	Primary	70%	10%	9/10 (90%)	Not Reported	3/10 (30%) Revision arm
Jaman et al. [16]	2019	Case Report	Iatrogenic ETSS	1	6 months	1/1 (100%)	Adjunct to repair	100%	0%	1/1 (100%)	Not reported	0/1 (0%)
Madeshiya et al. [10]	2024	RCT	Trauma 61.8% Rhinorrhea 38.2% Otorrhea	44 intervention (AZM) 32 Control (No AZM)	18 months	44/76 (57.8%)	Primary	Not Reported	Not Reported	Not Reported	15mg/kg	Not Reported
Leibu et al. [17]	2017	Retrospective	Trauma 32% Rhinorrhea 68% Otorrhea	54 Pediatric	4 months	54/54 (100%)	Primary	12.9%	0%	4/54	Not reported	Not reported
Abrishkamar et al. [18]	2013	RCT	Trauma 49.1% AZM initiated within first 48h 50.9% AZM initiated after first 48h	57	Not reported	57/57 (100%)	Primary	7.1% AZM<48h 31% AZM>48h	Not reported	1/28 (3.6%) 3/29 (10.3%)	25mg/kg/day	Not reported
Tilak et al. [8]	2019	Retrospective	Spontaneous CSF Rhinorrhea	16	470 days	16/16 (100%)	Primary	Not reported	Not Reported	11/16 (68.8%)	250mg BID	Not reported
Chaaban et al. [19]	2024	Prospective	Spontaneous, Iatrogenic, Traumatic	36	Not reported	36/36 (100%)	Adjunct to repair	5.6%	27.8%	36/36 (100%)	36/36 500mg Once 26/36 500mg BID	Not reported
Brainard et al. [20]	2012	Retrospective	Spontaneuous CSF Otorrhea	26	Not reported	3/26 (11.5%)	Adjunct to repair	0%	Not reported	26/26 (100%)	Not reported	Not Reported
Martinez-Capoccioni et al. [21]	2017	Retrospective	Spontaneous Rhinorrhea	35	8 months	35/35 (100%)	Adjunct to repair	100%	Not reported	35/35 (100%)	Not reported	1/35 (2.9%)
Jiang et al. [2]	2018	Retrospective	Spontaneous Rhinorrhea	48	1.5 years	19/48 (39.6%)	Adjunct to repair	27%	6.3%	48/48 (100%)	Not reported	6/19 (31.6%) AZM 3/29 (10.3%) No AZM
Chaaban et al. [5]	2014	Prospective	Spontaneous	46	93 weeks	23/46 (50%)	Adjunct to repair	91.3%	41.3%	46/46 (100%)	500mg	3/46 (6.5%) No AZM 1/46 (2.2%) AZM
Xie et al. [22]	2015	Retrospective	Spontaneous CSF Rhinorrhea	25	17 months	8/25 (32%)	Adjunct to repair	0%	4%	25/25 (100%)	1-2g/day	0%
Kreatsoulas et al. [1]	2020	Retrospective	Spontaneous CSF Rhinorrhea	46	15 months	12/46 (26.1%)	Adjunct to repair	0%	32.6% No AZM	46/46 (100%)	Not reported	2/46 (2.2%) No AZM
McCormick et al. [23]	2021	Retrospective	Spontaneous	55	12 months	36/55 (65.5%)	Adjunct to repair	100%	27.3%	55/55 (100%)	500 BID 32/36 500 TID 4/36	1/55 (1.8%)
Martinez-Capoccioni et al. [24]	2015	Retrospective	Spontaneous CSF Rhinorrhea	25	1 mo-6 years	25/25 (100%)	Adjunct to repair	100%	0%	25/25 (100%)	500 BID	1/25 (4%)
Aaron et al. [25]	2014	Prospective	Spontaneous	16	1 year	11/16 (68.8%)	Adjunct to repair	100%	25%	16/16 (100%)	500mg Once	Not reported
Woodworth et al. [11]	2008	Retrospective	Spontaneous	56	34 months	56/56 (100%)	Adjunct to repair	85.7%	23.2%	56/56 (100%)	500mg BID	6/56 (10.7%)
Hong et al. [26]	2021	Retrospective	Spontaneous	5	5.4 months	5/5 (100%)	Adjunct to repair	Not reported	40%	5/5 (100%)	Not reported	0/5 (0%)
Alkhotani [27]	2019	Case Report	Spontaneous CSF Rhinorrhea	1	6 months	1/1 (100%)	Adjunct to repair	0%	100%	1/1 (100%)	Not reported	0/1 (0%)
Matsubara et al. [28]	2014	Case Report	Spontaneous CSF Rhinorrhea	1	14 months	1/1 (100%)	Adjunct to repair	100%	0%	1/1 (100%)	Not reported	0/1 (0%)
Rosenfeld et al. [29]	2013	Case Series	Spontaneous CSF otorrhea (1) and rhinorrhea (3)	4	Not reported	2/4 (50%)	Adjunct to repair	Not reported	25% No AZM 25% AZM	2/4 (50%)	Not reported	Not reported
Al-Balushi et al. [30]	2023	Case Series	Spontaneous CSF Rhinorrhea	5	Not reported	3/5 (60%)	Adjunct to repair	Not reported	Not Reported	3/5 (60%)	Not reported	Not reported
Lin et al. [31]	2020	Case Report	Spontaneous CSF Rhinorrhea	1	8 months	1/1 (100%)	Adjunct to repair	100%	0%	1/1 (100%)	500mg BID	0/1 (0%)

Among this cohort, a total of 22.8% of patients required permanent CSF diversion. Notably, Kreatsoulas et al. reported that 32.6% of patients who did not receive acetazolamide required permanent CSF diversion, while none of those who received adjunctive acetazolamide required permanent CSF diversion following initial surgical repair [1]. CSF leak recurrence was not reported in five of the reviewed studies. When the results of the other 12 studies were pooled, 8.1% of those who received adjunctive acetazolamide went on to require reoperation for recurrence of CSF leak, while 7.8% of patients who did not receive adjunctive acetazolamide required reoperation for recurrence of CSF leak.

The most common side effects of acetazolamide use were nausea, dysgeusia, fatigue, weight gain, and paresthesias. Severe complications included hypersensitivity reactions and metabolic acidosis [25,26,29]. Additionally, acetazolamide use requires frequent monitoring of laboratory values to prevent electrolyte abnormalities. Often, management by a multidisciplinary team of neurology, neurosurgery, and neuro-ophthalmology is necessary when used long-term.

Discussion 

CSF leaks may occur from trauma, prior surgery of the skull base, or secondary to the chronic effects of IIH. Prompt intervention is needed to manage this condition definitively, and surgical repair or CSF diversion is often considered the most durable treatment modality [2,3,5]. Medical therapy with acetazolamide has been suggested as an alternative or adjunctive therapy to manage CSF leak, as it decreases CSF production and lowers intracranial pressure [7,8,32]. This may improve the efficacy of repair or even circumvent the need for surgical intervention in select patients. As minimally invasive or non-surgical approaches are often favorable, especially in patients with chronic medical conditions, it is important to highlight the utility of a medical strategy to better inform clinicians of alternative, nonsurgical options for patients with CSF leaks. This review aims to highlight the patients who may benefit from the addition of acetazolamide in the treatment of CSF leaks.

Acetazolamide has moderate success in treating patients with iatrogenic CSF leaks. Jamshidi et al. reported complete resolution of CSF leak from transsphenoidal surgery when patients were treated with high-volume lumbar puncture followed by acetazolamide [13]. Additionally, Phogat et al. suggested a similar approach to resolve CSF leaks following endoscopic endonasal surgery with no patients requiring permanent diversion; however, they did not report on the rate of leak recurrence [14,32]. In all of these groups, less than half of patients required permanent CSF diversion when they were initially treated with lumbar drainage. Jiam et al. utilized acetazolamide in only one patient who had a recurrent CSF leak after temporary diversion. The remainder of patients were treated with only temporary diversion. Comparatively, the rates of surgical repair were nearly eight-fold higher when compared to the studies that utilized acetazolamide and temporary diversion [15]. CSF leak was effectively treated when acetazolamide was combined with temporary CSF diversion; thus, it may be difficult to determine which modality was more effective in preventing the need for surgical repair and/or permanent diversion. Furthermore, the extent of the operative field, the method of skull-base defect repair, and individual patient factors may all contribute to the success of CSF leak repair [33,34]. In certain demographics, such as patients with higher preoperative intracranial pressure, acetazolamide may be highly beneficial when accompanied by temporary CSF diversion.

In the event of a traumatic CSF leak, acetazolamide appears to have a beneficial effect in preventing the need for more invasive treatment. Abrishkama et al. noted a three-fold and four-fold lower rate of surgical repair and lumbar puncture/drainage in the patients who received acetazolamide early in their treatment course [18]. While less than 10% of patients in the cohort received acetazolamide and temporary diversion, this indicates that there may be some benefit to early intervention with acetazolamide in patients with traumatic CSF leaks. However, the severity of trauma and mechanism of injury may impact the efficacy of treatment regardless of timing. Leibu et al. reported a period of observation followed by acetazolamide and CSF drainage in 54 patients who had early persistent CSF leaks. All patients who presented with early persistent CSF leak and received both acetazolamide and temporary CSF diversion had resolution of their CSF leak [17]. Interestingly, the only study that did not report a significant benefit of acetazolamide was from Madeshiya et al., who reported no significant difference in the duration of CSF leak in patients who received acetazolamide compared to the patients who were treated with observation, bed rest, and sedation [10].

Though spontaneous CSF leaks are typically treated with upfront repair by a multidisciplinary surgical team of otolaryngologists and neurosurgeons, there appeared to be a benefit with adjunct acetazolamide treamtent. Tilak et al. demonstrated there may be some utility for pre-treatment of CSF leaks with acetazolamide, as 5 out of 16 patients resolved with acetazolamide treatment alone, while the other 11 patients required further treatment with surgical repair [8]. As a whole, acetazolamide increased the success of surgical repair when given to patients who had known postoperative intracranial hypertension, often identified by lumbar puncture prior to surgical intervention [23,25,26]. However, as reported by others, despite surgical repair and acetazolamide therapy, reoperation and permanent CSF diversion were frequently required [1,5,19,23,25,26,29].

The size of study cohorts and timing of postoperative acetazolamide administration differed greatly between reviewed studies, which may have impacted its efficacy. Additionally, studies noted issues with medication compliance potentially confounding results. As such, the risks and benefits of long-term acetazolamide use must be weighed against the risks of surgical repair and permanent CSF diversion.

It is important to address several limitations of this study. (i) The articles reviewed had significant variability in their design. In addition, (ii) the indications for acetazolamide use, even outside of CSF leak, were not always clearly reported, and (iii) most of the studies included in this review were retrospective, allowing for the introduction of bias from the authors and reviewers.

## Conclusions

Acetazolamide should be considered as part of the treatment armamentarium of CSF leak. While surgical treatment is often necessary, acetazolamide therapy, in selected cases, may temporize or adjudicate the need for more invasive repair strategies. When informing patients of their treatment options, the traditional approaches of surgical repair or permanent CSF diversion are likely the most definitive forms of treatment. However, selecting the appropriate patients for medical therapy may provide some patients with an alternative to more invasive approaches with equal treatment efficacy.
